# Primary Lateral Patellar Dislocation Associated with an Increased Sulcus Angle in an African Child: A Case Report of Synthetic Graft-Assisted Medial Patellofemoral Ligament Reconstruction

**DOI:** 10.3390/reports9030232

**Published:** 2026-07-20

**Authors:** Kei Nagasaki, Manabu Mitsuhashi, Taketoshi Seino, Mizuki Toura, Yoshifumi Kudo

**Affiliations:** Department of Orthopedic Surgery, Showa Medical University, Tokyo 142-8666, Japan; manabu0913@med.showa-u.ac.jp (M.M.); notstraighter@gmail.com (T.S.); gm23-m029@med.showa-u.ac.jp (M.T.); kudo_4423@med.showa-u.ac.jp (Y.K.)

**Keywords:** medial patellofemoral ligament, sulcus angle, patellar dislocation, patellar instability, African child

## Abstract

**Background and Clinical Significance**: Patellar dislocation is considered uncommon in African populations, a finding often attributed to characteristically deeper femoral trochlear grooves that confer increased patellofemoral stability. Nevertheless, individual anatomical variations may predispose certain patients to instability despite population-based trends; **Case Presentation**: We report the case of a 12-year-old African pediatric patient who presented with a primary traumatic lateral patellar dislocation. Magnetic resonance imaging (MRI) demonstrated rupture of the medial patellofemoral ligament (MPFL) and a markedly increased sulcus angle of 159°, consistent with a shallow trochlear groove. The patient underwent MPFL reconstruction using a synthetic ligament to minimize the risk of physeal injury; **Conclusions**: This case shows the importance of individualized anatomical assessment in pediatric patellar instability. Even in populations generally considered to have lower anatomical risk, marked individual variation in trochlear morphology may influence treatment decisions. When surgical stabilization is selected in skeletally immature patients, physeal-sparing techniques should be carefully considered.

## 1. Introduction and Clinical Significance

Patellar dislocation is a relatively frequent injury among pediatric and adolescent patients, particularly during sports and high-impact physical activities [1]. The dislocation most commonly occurs in the lateral direction and is often associated with predisposing anatomical factors, including trochlear dysplasia, patella alta, an increased tibial tubercle–trochlear groove (TT–TG) distance, and generalized ligamentous laxity [2]. Among these factors, trochlear morphology has been consistently identified as a critical determinant of patellofemoral joint stability [3].

The sulcus angle, measured on axial imaging modalities such as magnetic resonance imaging (MRI) or computed tomography (CT), serves as an indirect indicator of trochlear depth. An increased sulcus angle reflects a flatter and shallower trochlear groove, which reduces patellar containment and increases the risk of lateral dislocation [4]. A sulcus angle exceeding 145° is generally regarded as abnormal and suggestive of trochlear dysplasia [5].

Several morphometric studies have reported population-level differences in trochlear and distal femoral morphology [6]. Some studies have suggested that individuals of African descent may demonstrate relatively deeper trochlear morphology and narrower sulcus angles compared with other populations [6]. However, these findings should be interpreted as population-level anatomical trends rather than definitive protection against patellar instability. Substantial individual variation may exist within any population [6].

This case report describes a 12-year-old African child with primary lateral patellar dislocation associated with a markedly increased sulcus angle. The significance of this case is not that patellar dislocation occurred in an African child, but that severe trochlear dysplasia was identified despite a demographic background generally associated in morphometric studies with deeper trochlear morphology. This case emphasizes that individual trochlear morphology should be assessed directly, even when population-level morphometric data suggest a lower risk profile.

## 2. Case Presentation

A 12-year-old African male presented with acute right knee pain and deformity following a traumatic lateral patellar dislocation sustained while playing soccer. The patient had no prior history of knee injury, recurrent instability, or generalized joint laxity. Physical examination revealed knee effusion, localized tenderness over the patella, and a positive patellar apprehension test. Standard radiographs showed no evidence of fracture or osteochondral injury; however, lateral patellar displacement was noted (Figure 1).

Closed reduction was performed immediately after presentation in the emergency department before MRI examination. Magnetic resonance imaging (MRI) of the right knee confirmed rupture of the MPFL (Figure 2) and demonstrated a sulcus angle of 159°, indicative of a markedly shallow femoral trochlear groove (Figure 3). The TT–TG distance was within the normal range (<20 mm), suggesting that trochlear morphology may have been the predominant anatomical risk factor in this case. Although conservative treatment is generally considered the standard initial approach for first-time traumatic patellar dislocation, surgical treatment was selected in this patient because of the combination of complete MPFL rupture, marked trochlear dysplasia with a sulcus angle of 159°, skeletal immaturity, and high functional demand related to sports activity. These factors were considered to increase the risk of recurrent instability, consistent with previous reports identifying young age and trochlear dysplasia as important risk factors for recurrence after first-time patellar dislocation [7].

Medial patellofemoral ligament reconstruction was performed using FiberTape^®^ suture tape and knotless SwiveLock^®^ suture anchors (Arthrex, Naples, FL, USA). A minimally invasive approach was used, requiring approximately a 2 cm incision on the femoral side and a 1 cm incision on the patellar side. Patellar fixation was achieved using suture anchors placed at the anatomical patellar attachment site. Femoral fixation was performed at the anatomical MPFL femoral insertion while carefully avoiding the distal femoral physis. No transphyseal drilling or bone tunnel creation was performed. Intraoperative assessment confirmed appropriate patellar tracking without excessive graft tension. Postoperative imaging confirmed appropriate patellar alignment (Figure 4), and the postoperative course was uneventful. A structured rehabilitation program was initiated, and the patient gradually returned to full physical activity within 6 months. No episodes of redislocation or subjective instability were reported during follow-up. At the 9-month follow-up, the patient achieved a Kujala score of 100, indicating favorable short-term functional recovery. Functional outcomes were assessed using the KOOS and Kujala scores (Table 1 and Table 2).

## 3. Discussion

The MPFL functions as the primary passive restraint against lateral patellar translation, particularly during the early phases of knee flexion [8]. In pediatric and adolescent patients, traumatic rupture of the MPFL frequently accompanies first-time lateral patellar dislocations [9,10]. Although conservative management may be appropriate for selected cases, surgical reconstruction is increasingly advocated in the presence of recurrent instability or high-risk anatomical features to restore patellofemoral stability and prevent subsequent dislocation episodes [11,12].

Trochlear dysplasia is among the most significant anatomical predictors of patellar instability [13]. The sulcus angle—defined by the intersection of the medial and lateral trochlear facets—serves as a quantitative measure of trochlear morphology [14]. Normal sulcus angles typically range from 125° to 145°, whereas increased values reflect a flattened trochlear groove and diminished patellar containment [14,15]. In the present case, a sulcus angle of 159° was identified, representing a markedly abnormal value and a structural predisposition to lateral patellar dislocation. Based on the available imaging findings, the trochlear morphology was classified as Dejour type A trochlear dysplasia, characterized primarily by a shallow trochlear groove without high-grade dysplastic features. In addition, the TT–TG distance was within the normal range, suggesting that trochlear morphology, rather than tibial tubercle lateralization, was the predominant anatomical factor contributing to instability in this patient [7,12,16].

The indication for surgical treatment after a first-time traumatic patellar dislocation remains controversial, particularly in skeletally immature patients [16]. Conservative treatment is generally recommended as the initial management strategy in most cases, especially when no osteochondral fracture or loose body is present [17]. However, treatment decisions should also consider the individual risk of recurrent instability. In the present case, MRI demonstrated complete MPFL rupture, and imaging showed marked trochlear dysplasia with a sulcus angle of 159°. In addition, the patient was skeletally immature and participated in sports activities. These combined factors were considered to increase the risk of recurrent instability.

Although no osteochondral fracture or loose body was identified, recurrent instability in this young and active patient was considered clinically relevant because repeated dislocation may lead to additional cartilage injury, persistent apprehension, activity restriction, and progressive patellofemoral dysfunction. Therefore, early surgical stabilization was selected as an individualized treatment option after considering the balance between the risk of recurrence and the invasiveness of surgery [18]. This decision should not be interpreted as a recommendation for routine surgical treatment after all first-time patellar dislocations.

Several morphometric studies have reported that individuals of African descent generally exhibit deeper trochlear grooves and narrower sulcus angles, which are features thought to confer enhanced patellofemoral stability [19]. Accordingly, the incidence of patellar dislocation is believed to be lower in this population [19]. However, the current case deviates from this population-based paradigm and highlights the limitations of relying on racial or demographic trends in clinical assessment. The presence of pronounced trochlear flattening in an African pediatric patient emphasizes the need for individualized anatomical evaluation rather than assumptions based on ethnicity alone.

This case implies the important role of advanced imaging in the assessment of patellar instability. MRI provides a comprehensive, noninvasive evaluation of trochlear morphology, sulcus angle, patellar alignment, and associated soft tissue injuries, including MPFL disruption [15]. Such detailed imaging is valuable for identifying high-risk anatomical features, guiding surgical decision-making, and optimizing operative planning, particularly in pediatric patients.

In this skeletally immature patient, FiberTape^®^ suture tape was selected to avoid donor-site morbidity associated with autologous tendon harvest and to allow reconstruction without transphyseal drilling [17]. Synthetic ligament reconstruction, including suture-anchor fixation techniques, has been reported in the literature, although most available evidence is derived from skeletally mature or recurrent-instability populations [20,21]. However, this evidence cannot be directly applied to patients with open physes. In the present case, the technique was modified to reduce the risk of physeal injury by using limited surgical exposure, suture-anchor fixation, and avoidance of femoral bone tunnel creation [22]. Therefore, this case should be regarded as an individual clinical experience rather than evidence supporting routine use of synthetic grafts in pediatric MPFL reconstruction.

The short-term postoperative findings in this patient included the absence of recurrent instability, resolution of pain, and improvement in functional scores. These findings suggest that synthetic graft-assisted MPFL reconstruction may be a treatment option in carefully selected skeletally immature patients. This report has several limitations. First, it describes a single patient, and the findings may not be applicable to all pediatric patients with patellar instability. Second, follow-up was limited to 9 months. Although no recurrent instability or major complications were observed during this period, a longer follow-up is needed to evaluate the long-term performance of synthetic graft reconstruction in growing children. Complications such as graft elongation, foreign-body synovitis, anchor-related osteolysis, growth disturbance, and postoperative stiffness may not become apparent until later. Continued clinical and radiographic follow-up is therefore important. Additional reports with longer follow-up will help determine the role of synthetic grafts in pediatric MPFL reconstruction.

This case indicates several important considerations: (1) the necessity of individualized radiologic assessment rather than reliance on population-based anatomical assumptions; (2) the value of MRI in identifying high-risk structural abnormalities; and (3) the feasibility of synthetic graft use in pediatric MPFL reconstruction as a means of preserving physeal integrity. Further research investigating anatomical variability among individuals may improve risk stratification and treatment planning for patellar instability.

## 4. Conclusions

This case report describes primary lateral patellar dislocation in an African child with a markedly increased sulcus angle. Despite reports suggesting that deeper trochlear morphology is more common in African populations, substantial individual variation can occur. In the present patient, short-term follow-up showed restoration of patellar stability and improvement in clinical outcomes after MPFL reconstruction using a synthetic ligament. However, longer follow-up is necessary before any conclusions can be drawn regarding the long-term performance of this technique in skeletally immature patients.

## Figures and Tables

**Figure 1:**
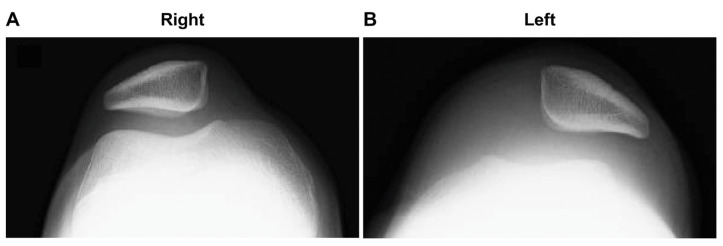
Plain radiographs of the right (**A**) and left (**B**) knees obtained at the time of injury. Anteroposterior and lateral views of the right and left knees show no evidence of fracture or osteochondral injury. The right knee demonstrates lateral displacement of the patella, suggesting acute lateral patellar dislocation and possible injury to the medial patellofemoral ligament.

**Figure 2:**
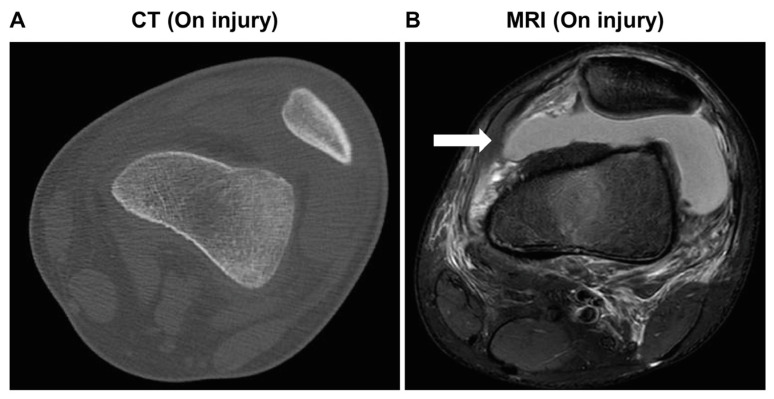
Preoperative imaging findings of the right knee. Computed tomography (CT) (**A**) and magnetic resonance imaging (MRI) (**B**) obtained at the time of injury. CT (**A**) demonstrates abnormal trochlear morphology, while the arrow in MRI (**B**) confirms rupture of the medial patellofemoral ligament.

**Figure 3:**
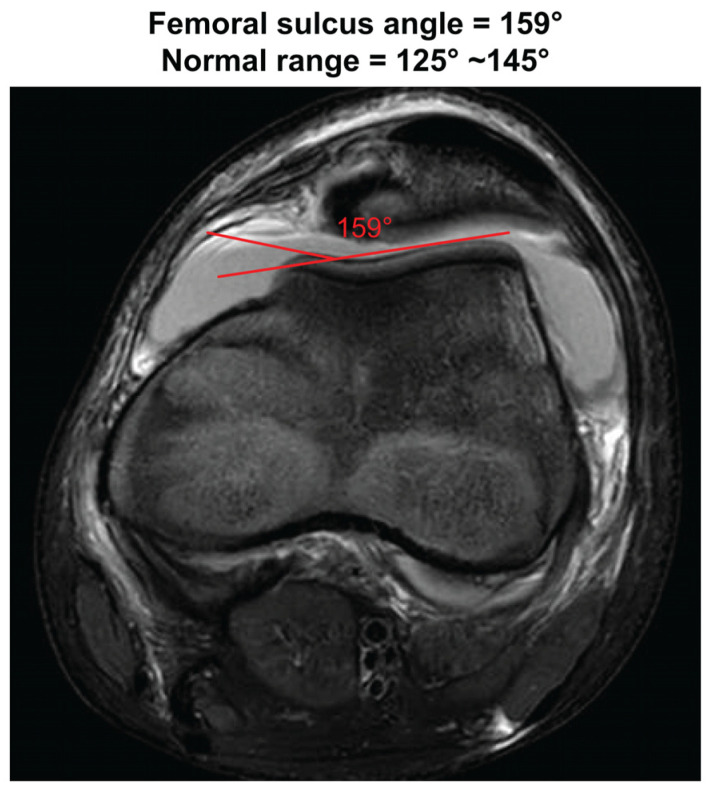
Measurement of the femoral sulcus angle on axial magnetic resonance imaging. The sulcus angle measured 159°, indicating a markedly shallow femoral trochlear groove. The normal reference range is 125–145°.

**Figure 4:**
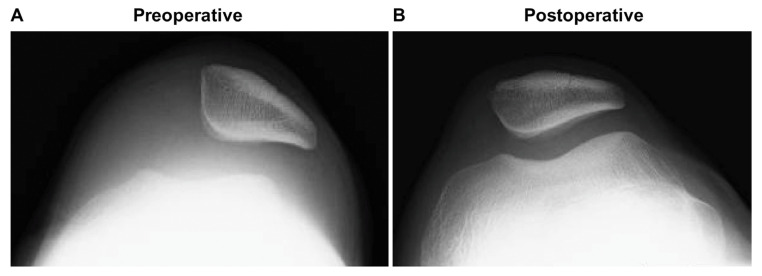
Preoperative (**A**) and postoperative (**B**) radiographic images of the right knee. The preoperative image is shown again to allow direct comparison with the postoperative radiograph. Comparison demonstrates restoration of appropriate patellar alignment following medial patellofemoral ligament reconstruction.

**Table 1:** Changes in Knee Injury and Osteoarthritis Outcome Subscale Scores.

Subscale	Preoperative	Postoperative (1 Month)	Postoperative (3 Months)	Postoperative (6 Months)	Postoperative (9 Months)
Symptoms	32	71	75	96	100
Pain	36	75	78	100	100
ADL	44	84	85	100	100
Sport/Rec	5	25	85	100	100
QOL	38	69	75	100	100
Total	31.0	64.8	79.6	99.2	100.0

KOOS: Knee Injury and Osteoarthritis Outcome Score; ADL: activities of daily living; Sport/Rec: sport and recreation; QOL: quality of life. The KOOS includes five subscales. Each subscale is scored on a scale from 0 to 100, with higher scores indicating better knee function. “Total” represents the mean of the five KOOS subscale scores (Symptoms, Pain, ADL, Sport/Rec, and QOL).

**Table 2:** Changes in Kujala Anterior Knee Pain Scores.

Parameter	Preoperative (On Injury)	Postoperative (1 Month)	Postoperative (3 Months)	Postoperative (6 Months)	Postoperative (9 Months)
Limp	3	3	5	5	5
Support	3	3	5	5	5
Walking	2	5	5	5	5
Stair climbing	10	8	8	10	10
Squatting	0	4	4	5	5
Running	0	0	8	10	10
Jumping	0	0	7	7	10
Prolonged sitting with knee flexion	6	8	8	10	10
Pain	3	8	10	10	10
Swelling	0	8	8	8	10
Painful patellar movement	4	6	6	10	10
Thigh muscle atrophy	3	5	5	5	5
Flexion difficulty	3	5	5	5	5
Total	37	63	84	95	100

Kujala score: This is a disease-specific outcome measure for patellofemoral disorders, ranging from 0 to 100 points, with higher scores indicating better knee function.

## Data Availability

The data supporting the findings of this study are not publicly available due to patient privacy and ethical restrictions but are available from the corresponding author upon reasonable request.

## Abbreviations

The following abbreviations are used in this manuscript:

MPFL	Medial patellofemoral ligament
MRI	Magnetic resonance imaging
CT	Computed tomography
KOOS	Knee Injury and Osteoarthritis Outcome Score
ADL	Activities of daily living
QOL	Quality of life
